# Radiotherapeutic Treatment of an Epidermoid Tumor in a Patient With Zanubrutinib-Treated Mantle Cell Lymphoma: The First Report of Concomitant Treatment

**DOI:** 10.7759/cureus.73378

**Published:** 2024-11-10

**Authors:** François Fabi, Louis-Philippe Grenier, Robert Delage, André Fortin

**Affiliations:** 1 Radiation Oncology, Centre intégré de cancérologie (CIC) Hôpital de l’Enfant-Jésus, Centre Hospitalier Universitaire de Québec - Université Laval (CHU de Québec-Université Laval), Québec, CAN; 2 Pharmacy, Centre Intégré de Cancérologie (CIC) Hôpital de l’Enfant-Jésus, Centre Hospitalier Universitaire de Québec - Université Laval (CHU de Québec-Université Laval), Québec, CAN; 3 Hematology and Oncology, Centre intégré de cancérologie (CIC) Hôpital de l’Enfant-Jésus, Centre Hospitalier Universitaire de Québec - Université Laval (CHU de Québec-Université Laval), Québec, CAN

**Keywords:** btki, concomitant treatment, mcl, radiotherapy, zanubrutinib

## Abstract

Mantle cell lymphoma (MCL) is a challenging B-cell non-Hodgkin lymphoma with a poor prognosis and frequent relapses. While treatment advancements such as rituximab and Bruton tyrosine kinase inhibitors (BTKi) like ibrutinib have improved outcomes, novel treatments are continually sought. Zanubrutinib, a second-generation BTKi, promises reduced side effects due to its high selectivity and reduced off-target inhibition. This paper presents a novel case of simultaneous radiotherapy and zanubrutinib treatment in a patient with MCL.

We describe a 76-year-old female with a history of MCL treated with zanubrutinib. The patient presented with a metastatic lesion in the parotid gland, which emerged from a previously treated spinocellular carcinoma. She underwent parotidectomy followed by adjuvant radiotherapy while continuing zanubrutinib. The combination was well-tolerated with minimal side effects, including grade 1 dermatitis and grade 2 mucositis, neither of which progressed significantly. Hematological parameters monitored during treatment showed delayed, transient lymphopenia and neutropenia, which resolved post-therapy. No dose adjustment was necessary.

The safety and efficacy of concurrent radiotherapy and zanubrutinib treatment in MCL patients are largely unexplored in clinical literature. This case represents the first documented instance of concurrent radiotherapy and zanubrutinib treatment. Our case suggests a favorable safety profile with manageable adverse effects, contrasting with concerns about increased toxicity with other tyrosine kinase inhibitors and radiotherapy combinations. These results indicate the feasibility of this approach with minimal adverse effects. Future studies should explore the broader applicability and efficacy of this treatment strategy, focusing on long-term outcomes and the interplay between BTKi therapy and radiotherapy response.

## Introduction

Mantle cell lymphoma (MCL) is an aggressive B-cell non-Hodgkin lymphoma (NHL), representing 5-7% of all lymphomas and 3-10% of all adult-onset NHLs [1,2]. Considered incurable, its clinical course is characterized by frequent relapses, which translates to a poor prognosis. The median survival for MCL is three to six years, although steady improvements have been made since the 1990s owing to the introduction of targeted therapies such as rituximab [2]. The development of orally available Bruton tyrosine kinase inhibitors (BTKi) represents another major clinical advance. Ibrutinib, the first-in-class compound, is now the preferred option for second-line therapy of relapsed-refractory MCL [1].

Zanubrutinib is a highly selective, irreversible second-generation BTKi. Compared to older-generation BTKis, its reduced off-target inhibition of Tec kinase in platelets and epidermal growth factor receptor (EGFR) appears to reduce the incidence of atrial fibrillation (AF), bleeding, rash, and diarrhea [3]. However, this novel agent has been found to induce higher rates of neutropenia [4].

The risk of disease flare-ups has been extensively described and discussed in the context of BTKi discontinuation in chronic lymphocytic leukemia (CLL). Such complications are also an important concern in MCL [5], and interruption of these systemic, continuous treatments is thus considered hazardous. In large part due to the improved pharmaceutical armamentarium, MCL patients now experience longer remissions and undergo continuous treatment for extended periods of time. However, MCL patients are at heightened risk of developing secondary malignancies (SM), particularly ENT and dermatological malignancies. Radiotherapy is a core therapeutic modality for solid lesions, including secondary malignancies. Concurrent radiotherapy during continuous ibrutinib treatment for CLL has been recently reported. While most patients did not experience severe adverse events, grade 3 or 4 dermatitis, neutropenia, and thrombocytopenia were reported [6]. However, no such data are available for the new-generation BTKi. To our knowledge, no clinical trial permits concurrent radiotherapy with zanubrutinib treatment. Moreover, the few reports that have investigated zanubrutinib treatment with radiotherapy have provided such treatments sequentially, never in combination [7-9]. The safety profile of such concomitant treatment thus remains undetermined.

Our work is, to our knowledge, the first report of simultaneous radiotherapeutic and zanubrutinib treatment and contributes to the limited literature examining this concomitant treatment modality.

## Case presentation

The patient was a 76-year-old woman with a history of hypertension, dyslipidemia, AF, and Paget disease. She was diagnosed 14 years earlier with a CD19+, CD5+, CD23+, SOX11- MCL with leukemic expression. She remained asymptomatic for eight years until she presented with anemia (106 g/L) and progressive leukocytosis (WBC 210 x 10^9^/L). At that time, the tumor board recommended four cycles of bendamustine-Rituximab. She remained asymptomatic for another four years until progressive leukocytosis and significant, widespread adenopathies were detected on imaging. Systemic treatment with zanubrutinib (320 mg daily) was initiated, driven by the reduced bleeding risk of zanubrutinib compared to ibrutinib in this patient, who was already on apixaban therapy due to her CHADS3 AF.

Zanubrutinib was well tolerated, and subsequent imaging revealed a significant reduction in lymph node involvement both supra- and infra-diaphragmatically. However, a new right-sided parotid mass was identified. A parotidectomy revealed a T0pN3b trifocal epidermoid carcinoma, with the largest focus measuring 3.2 cm. Perineural invasion was noted, and post-surgical margins were positive for two foci. The patient was referred for adjuvant radiotherapy. Lymph nodes were negative, and it was determined that the masses were metastatic lesions from a previously treated spinocellular carcinoma treated two years prior. An 18FDG-PET scan showed a 1.3 cm hypermetabolic lesion at the surgical site. A total of 68.8 Gy was delivered to the hypermetabolic focus and 60 Gy to the gland over 32 fractions.

Due to the risk of disease flare-ups and following recent case reports supporting the safety of ibrutinib during radiotherapy, zanubrutinib treatment was continued throughout the six weeks of radiation. However, data regarding the safety profile of combining radiotherapy with zanubrutinib were unavailable. The patient was monitored weekly by both the radiation oncology and hematology-oncology teams. The treatment was well tolerated, with no dose adjustments required during radiotherapy. Grade 1 radiation dermatitis was noted early, along with a 1.5 cm grade 2 radiation-induced mucositis inside the patient’s cheek. Neither condition progressed. While the mucositis resolved quickly, the radiation dermatitis persisted for three months post-treatment.

Table 1 summarizes hematological parameters before, during, and after radiotherapy with zanubrutinib. The patient experienced transient grade 2 lymphopenia toward the end of the combination treatment, which partially resolved. She also developed progressive neutropenia, reaching grade 3 shortly after completing treatment, but subsequently recovered within weeks. No infectious symptoms or fever were reported. No progression of MCL was observed on subsequent PET imaging. Unfortunately, disease recurrence was detected three months later. No further radiotherapeutic interventions are planned, and she was referred to the palliative care unit for specialized pain management. She continues to be on zanubrutinib without signs of MCL recurrence.

**Table 1:** Hematologic parameters before, during, and after radiotherapy with zanubrutinib

Parameters	Zanubrutinib			Radiotherapy + Zanubrutinib		Zanubrutinib	
	Initiation	6 weeks	3 months	D15	D37	2 weeks post-RT	3 months post-RT
Leucocytes (x 10^9^/L)	18.9	40.8	7.6	4.2	3.4	3.3	4.1
Neutrophils (x 10^9^/L)	3.7	4.2	3.3	2.9	1.9	0.6	3.5
Lymphocytes (x 10^9^/L)	14.5	35.1	3.8	0.8	0.5	0.8	0.4
Platelets (x 10^9^/L)	98	102	126	155	130	139	159
Hemoglobin (g/L)	130	118	144	131	126	131	136

## Discussion

Ibrutinib demonstrated great efficacy in a phase II study as a single agent in relapsed/refractory B-cell malignancies, with a 68% overall response rate (ORR), including 21% complete responses after a median follow-up of 15.3 months [10]. Subsequently, the ASPEN study confirmed the generally improved side effect profile of zanubrutinib, although all-grade neutropenia was reported [4]. In 2019, the FDA granted accelerated approval for this drug in relapsed/refractory MCL following evaluation of the BGB-3111-206 phase II open-label study. The ORR was 83.7%, and median progression-free survival was 33 months after a median follow-up of 35.3 months [11]. Such results have propelled these compounds to the forefront of MCL management. Consequently, we can anticipate a growing number of patients receiving BTKi therapy in the future, with longer durations under treatment. It thus appears reasonable to inquire about the safety of concomitant radiotherapy, especially considering that treatment interruption is associated with rapid relapse.

In this case, the patient tolerated the combined treatment well. Minimal side effects were observed, with only transient neutropenia occurring, which had no associated clinical complications. The adverse effect profile of combined ibrutinib with radiotherapy has been studied by Ho and colleagues [6]. Their data suggest that concurrent ibrutinib and radiotherapy increase the risk of grade 3 or higher neutropenia compared to sequential administration, aligning with our experience. The concurrent use of various tyrosine kinase inhibitors (TKis) and radiotherapy has also been previously studied. A 2021 meta-analysis showed no enhancement in efficacy, and the study raised concerns about increased toxicity [12]. Indeed, a higher incidence of grade 3 toxicity was found, potentially due to a radiosensitizing effect of such compounds [13]. However, while no BTKis were included in this analysis, the results can still be informative in our context. Earlier-generation BTKis, such as ibrutinib, are well-known to exhibit off-target inhibitory effects on EGFR and other kinase-dependent signaling pathways. While these off-target effects are reduced, they remain an issue in next-generation BTKis such as zanubrutinib [14]. Alternatively, concurrent radiotherapy was reported as feasible, with an acceptable increase in toxicity when combined with BRAF inhibitors dabrafenib and vemurafenib [15]. Nevertheless, the publication of two case reports of radiation necrosis associated with vemurafenib use and closely sequenced central nervous system irradiation suggests that caution is still advised [16]. To date, the effect of BTKis on radiosensitivity has not been extensively studied. Given their imperfect specificity, some of the results obtained in the context of TKIs may translate to their use. In vitro data have shown that ibrutinib radiosensitizes pancreatic cancer cells through EGFR and PI3K/Akt pathway inhibition [17]. While no such information is available for zanubrutinib, we can expect this effect to be reduced, considering the improved off-target profile of this next-generation drug.

Regarding the efficacy of this combined approach, it is difficult to draw any firm conclusions. In this specific case, the patient experienced early local recurrence of their solid tumor, but control of the MCL was retained. We can surmise that the immunomodulatory effect of BTKis [18] could alter radiotherapy effectiveness, particularly as their intricate relationship is emerging as a core driver of therapeutic response [19]. T-cell response is known to be instigated and potentiated by radiation, an effect that could be blunted by BTKi immunomodulatory effects, such as dysregulation of Th17/Treg differentiation [20]. Unfortunately, to our knowledge, no available data investigates the mechanistic or clinical effect of BTKis on radiotherapy.

## Conclusions

This case report presents an encouraging example of the potential for concurrent radiotherapy and zanubrutinib treatment in MCL, offering a pathway to explore the feasibility of this combination. The patient tolerated the treatment well, experiencing only mild, manageable side effects such as grade 1 dermatitis, grade 2 mucositis, and transient neutropenia, all of which resolved post-therapy. These findings highlight zanubrutinib’s promise as a viable option for patients requiring continuous BTKi treatment during radiotherapy.

While this single case provides a foundation, more extensive research is necessary to validate these findings across a larger cohort of MCL patients and to examine long-term outcomes. Future studies should aim to characterize the mechanistic effects of zanubrutinib on radiosensitivity and its potential impact on both MCL control and secondary malignancy recurrence. Understanding the interplay between BTKi therapy and radiotherapy response will be essential in guiding safe, effective treatment protocols, ultimately improving patient management in cases where concurrent therapy is required. This case thus lays the groundwork for broader investigations into BTKi and radiotherapy combinations in MCL and other lymphomas.
